# Adsorption of Pollutants from Colored Wastewaters after Natural Wool Dyeing

**DOI:** 10.3390/ma15041488

**Published:** 2022-02-16

**Authors:** Alenka Ojstršek, Primož Vouk, Darinka Fakin

**Affiliations:** Faculty of Mechanical Engineering, University of Maribor, Smetanova 17, 2000 Maribor, Slovenia; vouk.primoz@gmail.com (P.V.); darinka.fakin@um.si (D.F.)

**Keywords:** wool dyeing, natural dyes, wastewater treatment, decoloration, heavy metals’ reduction, adsorption

## Abstract

The presented study assesses the efficiency of selected adsorbents, zeolite 4A in two particle sizes and pelletized activated carbon (AC), for the potential removal of color, chemical oxygen demand (COD), total organic carbon (TOC) and metals from wastewaters after natural wool dyeing. Firstly, the natural coloring compounds were extracted from dried common walnut (*Juglans regia*) leaves and used further for exhaustion dyeing of wool fibers, together with three different metallic salts in two concentrations (meta-mordanting). Effluents with higher mordant concentration were additionally treated according to a shake-flask adsorption experiment. The obtained results revealed efficient removal of exceeded metallic ions by zeolite (up to 94.7%), on account of their superior ion exchange capability as compared to AC. The zeolites also reduced turbidity and electrical conductivity significantly. On the other hand, AC was more efficient for the reduction in organic pollution, COD up to 96% and TOC up to 95%, due to its higher specific surface area and total pore volume, and, thus, higher potential for adsorption of different compounds in comparison to 4A. All three proposed adsorbents lowered wastewaters’ coloration remarkably, up to 78% (AC) and up to 71% (4A), depending on the type of effluent/mordant and inspected wavelength; although, the spectral absorbance coefficient (SAC) values remained highly above the limit values for discharge of wastewaters into watercourses.

## 1. Introduction

Over the past decade, there has been a growing demand for eco-friendlier, non-toxic coloring compounds, with fewer negative effects on the organism, especially for health-sensitive applications such as the coloration of food, drugs and cosmetics, and dyeing of textiles for children [1]. Natural color-possessed compounds can be derived by the extraction of numerous plants/plant parts (wood, roots, stems, branches, leaves, flowers, fruits, seeds and peels), invertebrates or minerals for the dyeing of various natural fibers [2]. In addition to coloration, natural dyes can add some additional (multi)functionalities to textile material, such as antibacterial, antioxidant, UV protection and insect repellent properties, widening their application purposes [3,4]. In spite of some advantages of natural dyes over synthetic, e.g., biodegradability, low toxicity, non-carcinogenicity, non-allergic, etc., they provide a lesser amount of coloring component (lighter color shades), have poor or no affinity towards the textile substrate, a narrower color range, difficulties in the shades’ reproducibility and inferior fastness properties, which have restricted their potential in the industrial-scale applications [5,6].

With the aim to increase the color depth and fastness properties, different metal mordants are usually employed, such as copper, chromium, tin, zinc and aluminum salts, leading to the disposal of unfixed toxic metals into the environment after dyeing [7]. Despite mordanting, a substantial quantity of coloring compounds remains unexhausted after dyeing, increasing the water’s coloration and turbidity significantly and, consecutively, reducing the transparency of water sources and affecting the photosynthesis rate of aquatic life [8]. Moreover, natural dyes are of organic origin and, thus, enlarge the total organic carbon (TOC) and chemical oxygen demand (COD) in colored wastewaters. The intensity of the color is changed by changing the pH during extraction of the same plant [9]. Furthermore, the addition of chemicals (acid or base) is inevitable during natural dyeing of protein- or cellulose-based textiles, influencing the pH of the effluent. Therefore, such kinds of wastewaters, when produced to a large extent, need to be handled properly before discharge into the environment.

Dias et al. [10] reported the kinetic and equilibrium study of natural annatto dye adsorption by kaolin. Sharma et al. [3] reused the wastewater of a henna dyebath three times (four dyeing cycles) for the coloration and functional finishing of linen fabrics, with a lower color strength of dyed samples after each dyeing. A similar approach was utilized in [7], where extracts from two fruits *Terminalia arjuna* and *Thespesia populnea* were reused for a second cycle of silk dyeing. The comprehensive study of madder dyebath’ wastewater performed by [11] indicated that the chemical components of the initial dye solution were different from those remaining in the exhausted dye solution. Therefore, he proposed a reconstruction of reused wastewater by the addition of a fresh coloring compound (in his study a madder) and water, to obtain the same quality of samples’ coloration. Instead of reusing the dyebath, some researchers employed plasma [12] or ultrasound [13] pre-treatment of textiles to increase the sorption of natural dyes on the surface, and some applied ultrasound during extraction/dyeing to enhance the amount of coloring compounds in the dyebath/on textiles [7,13]. Different studies have dealt with the replacement of toxic metal mordants with more environmentally friendlier ones, i.e., tannin [4], myrobalan (*Terminalia chebula*) [7], extract of acacia, henna, turmeric, and pomegranate bio-mordants [13].

Compared to the extensive studies of the reducing of synthetic dyes from wastewaters and, consecutively, the numerous treatment techniques proposed [8,14,15,16,17,18,19], studies performing the treatment of colored wastewaters extracted from natural sources are almost neglected by the researchers. In addition, articles addressing the environmental aspect of the application of natural dyes are very limited. Handayani et al. [20] examined the characteristics of wastewater collected from a batik home-based industrial dyeing by a natural dye extract from mahogany (*S. macrophylla*), indigo (*I. tinctoria*), and myrobalan (*T. bellirica*). They found that the concentration of total suspended solids (TSS), biological oxygen demand (BOD_5_) and COD exceeded the Quality Standard for Textile Effluent set by The World Bank. Moreover, a BOD_5_/COD ratio below 0.3 implied that the wastewater was not subjected to biodegradation. Similar results were gained by Muniz dos Santos Silva [21], analyzing effluents from natural dyeing of cotton and wool with *Croton urucurana* Baill. bark. They also found out that Al^3+^ and Fe^2+^ ions in effluents exceed the limit values for discharge in watercourses highly when these metals were used as mordants.

Common walnut (*Juglans regia*) is a deciduous tree of the *Junglandaceae* species widely cultivated across Europe [6]. Its leaves contain a large amount of phenolic compounds, i.e., naphthoquinones and flavonoids, which are a source of a plant-based reddish-brown natural dye.

Adsorption is a well-known, simple, easy to operate, low-cost, and effective purification technique for the removal of coloring compounds and metal ions from diverse wastewaters [22,23]. Different parameters affect the adsorption efficiency, such as the physical or chemical properties of the adsorbent, the surface area/unit weight of the adsorbent, and the nature of the compound being adhered on the surface of the adsorbent.

Based on the above-discussed, the aim of this research was to evaluate the adsorption ability of three selected adsorbents to reduce color, TOC, COD and ions of heavy metals from model wastewaters generated after wool dyeing utilizing common walnut leaves’ extract and three different mordants. According to our knowledge, there is no literature regarding the use of the proposed adsorbents, i.e., zeolite 4A (in two different granule sizes) and activated carbon, for the treatment of wastewaters after wool dyeing with natural dyes, a fact which manifests the novelty of this work.

## 2. Materials and Methods

### 2.1. Materials

Experiments were conducted on yellowish-colored wool fibers obtained from Soven, d.o.o. (Selnica ob Dravi, Slovenia). The source materials were washed at 40 °C for 20 min employing a neutral non-ionic washing agent, with the aim to eliminate natural impurities and industrially applied additives. Washed samples were further rinsed in warm and cold water and dried at a temperature of 60–70 °C and, after that, kept at constant conditions (temperature of 20 ± 2 °C and relative humidity of 65 ± 5%).

**Figure 1:**
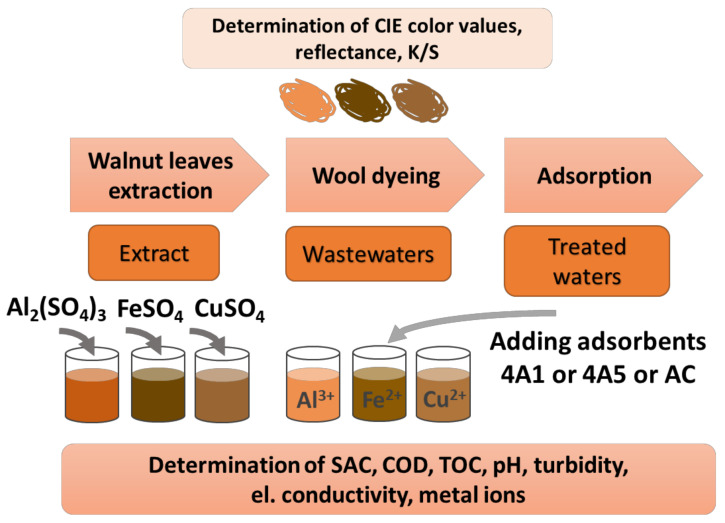
Scheme of the experimental work.

Aluminum sulfate octadecahydrate (Al_2_(SO_4_)_3_·18H_2_O), ferrous sulfate heptahydrate (FeSO_4_·7H_2_O), copper sulfate pentahydrate (CuSO_4_·5H_2_O), hydrochloric acid (HCl), nitric acid (HNO_3_) and acetic acid (CH_3_COOH) were analytically graded reagents obtained from Merck KGaA, (Darmstadt, Germany). Zeolite 4A in a granulated form of two different sizes, 1–3 (4A1) and 3–5 mm (4A5), were synthesized/granulated and kindly supplied by Silkem Zeolite Production, Inc. (Kidričevo, Slovenia). Pelletized activated carbon (AC) with particles’ size of Φ4 mm was purchased from Riedel de Haen. Before the adsorption trials started, all adsorbents were rinsed with deionized water and dried at a temperature of 105 °C for 3 h, followed by condensation at constant conditions for 24 h. The phases of the proposed experimental work are presented in Figure 1.

### 2.2. Preparation of Model Wastewaters

#### 2.2.1. Extraction of Walnut Leaves

Fresh leaves of a walnut tree were collected during the spring of 2021 in the eastern part of Slovenia at an altitude of 220 m above sea level. They were separated from the stalks, dried and chopped into small pieces. The as-prepared leaves were extracted according to a previously optimized procedure [6]. Briefly, the extraction was accomplished in deionized water at boiling temperature on a hot plate for 1 h, using a liquor-to-weight ratio of 1:20 (300 g of plant material against 6 L of deionized water). Afterward, the extraction mixture (the extract together with a plant material) was kept at room temperature for approximately 24 h, and then filtered for trial purposes.

#### 2.2.2. Exhaust Dyeing

Firstly, six dyebaths were prepared by admixing of 600 mL of walnut leaves’ extract and an individual mordant, i.e., Al_2_(SO_4_)_3_·18H_2_O, FeSO_4_·7H_2_O and CuSO_4_·5H_2_O in two concentrations, 3 and 6 g/L. Then, the wool fibers were dyed according to the exhaustion dyeing procedure (meta-mordanting) by means of a laboratory device, Ahiba (Werner Mathis AG, Oberhasli, Switzerland), using a medium bath agitation [6]. Dyeing was started at a temperature of 20 °C, when 10 g of wool was put into the extracted baths, and the pH was adjusted to 4 by CH_3_COOH, raising the dyebath’s temperature to 100 °C with a heating rate of 2 °C/min, thereon being maintained at 100 °C for 45 min, and then lowered to 70 °C with a cooling rate of 3 °C/min. The dyed fibers were taken from the baths, rinsed in warm and cold water and after that dried at an ambient temperature. Three residual bath effluents with 6 g/L of initial mordant concentration were characterized in terms of pollution parameters’ determination and preserved further for the adsorption trials.

### 2.3. Batch Adsorption Trial

A batch trial was employed with the aim of treating wastewaters from natural wool dyeing utilizing adsorption. A pre-optimized quantity (20 mg) of an individual adsorbent was put into an Erlenmeyer flask of 250 mL, together with 100 mL of an individual source of wastewater, without further modification in terms of pH changes or electrolyte addition. All flasks were sealed and shaken on an orbital shaker with 160 rpm for 24 h at a room temperature of 22 ± 2 °C. The batch experiments were performed in duplicate. Afterward, the adsorbents were taken out from the flasks and further characterized.

### 2.4. Analytical Procedure

#### 2.4.1. Characterization of the Dyed Fibers

Reflectance measurement of the dyed samples was accomplished within a spectral range of 400–700 nm wavelengths, by means of a two-ray spectrophotometer, Spectraflash SF600 Plus (Datacolor, Luzern, Switzerland), at a standard illuminant D65 (LAV/Spec. Incl., d/8, D65/10°), from which the CIE color values were calculated using the Datacolor QC 600, V3.3 software.

In addition, the relative color strength (K/S) of samples was calculated over the whole visible spectrum (400–700 nm), by means of the following Kubelka–Munk Equation (1):

(1) $$K/S=\frac{{\left(1-R\right)}^{2}}{2R},$$

where K is the absorption coefficient; S is the light-scattering coefficient; and *R* is the decimal fraction of the colored sample’s reflectance.

#### 2.4.2. Analysis of Adsorbents

The surface morphology of the employed adsorbents was investigated employing Scanning Electron Microscopy (SEM). An individual granulated adsorbent was crushed into a powder form and placed on an adhesive carbon tape attached to a brass holder and inspected using a Gemini Supra 35 V P Scanning Electron Microscope (Carl Zeiss NTS GmbH, Oberkochen, Germany), with a maximum scan resolution of up to 1.5 nm at 20 kV. With the aim to determine the functional groups of adsorbents, Fourier Transform InfraRed (FTIR) spectroscopic measurement was performed using an FTIR System Spectrum GX spectrophotometer (Perkin Elmer) with a Golden Gate ATR attachment and a diamond crystal. The transmittance spectra were gained in the range 4000–650 cm^−1^, by selecting 16 scans and a resolution of 4 cm^−1^. The surface physical proprieties of the adsorbents were characterized with a Tristar 3000 adsorption analyzer (Micromeritics, Unterschleissheim, Germany) at 77 K. Prior to the measurement, the samples were purged with nitrogen overnight at 200 °C. The BET specific surface area was calculated using the adsorption branch of the isotherm in the relative pressure (p/p_0_) ranging between 0.01 and 0.1.

#### 2.4.3. Wastewaters’ Analysis

Liquid samples for analyses were taken before dyeing—extract (E-Al, E-Fe, E-Cu), after dyeing—wastewater (W-Al, W-Fe, W-Cu), and after treatment with three different adsorbents (4A1-Al, 4A1-Fe, 4A1-Cu, and 4A5-Al, 4A5-Fe, 4A5-Cu, and AC-Al, AC-Fe, AC-Cu); altogether 15 samples. All samples were characterized by measuring the absorbance at wavelengths of 436, 525 and 620 nm according to Standard EN ISO 7887:2012, using a special measuring probe (10 mm optical length) on a Cary 60 UV/Vis spectrophotometer (Varian, Columbus, OH, USA). Prior to absorbance measurement, all liquid samples were centrifuged for 15 min at 3000 rpm in order to exclude turbidity. In addition, the Spectral Absorption Coefficient (SAC) was calculated (in·m^−1^) as a quotient between the measured absorbance at a defined wavelength and the optical pathway (in mm), multiplied by a factor of 1000 (unit conversion).

The pH was measured according to the ISO 10523 Standard by means of an MA 235 portable pH meter (Mettler Toledo). The Total Organic Carbon (TOC) was determined using a Multi N/C 2100 S analyzer (Analytik Jena, GmbH, Jena, Germany) in accordance with the ISO 8245 Standard, and the Chemical Oxygen Demand (COD) in accordance with the ISO 6060 Standard (titration method). The electrical conductivity was determined by a Conductivity Meter (Mettler Toledo, Columbus, Ohio, USA) according to Standard EN 27888, and turbidity by a TB1 portable turbidimeter (VELP Scientifica Srl, Usmate Velate, Italy) according to Standard ISO 7027 1:2017.

Al^3+^ metal ions were determined by means of an Analyst 600 Atomic Absorption Spectrometer (Perkin Elmer, Waltham, MA, USA) with a hollow cathode lamp as the radiation source and a deuterium background corrector at a wavelength of 309.3 nm, and Fe^2+^ and Cu^2+^ ions by an AA240 Atomic Absorption Spectrometer (Agilent, Santa Clara, CA, United States) at wavelengths of 248.3 and 324.8 nm, respectively. The operating conditions adjusted in the spectrometer were carried out according to the standard guidelines of the manufacturer. Before metal ions’ determination, 5 mL of each individual sample was diluted in 5 mL of concentrated HNO_3_ and filled up to 50 mL with MilliQ water. Each measurement was performed in duplicate.

The percentage of metal ions, COD and TOC reduction I was calculated in accordance with Equation (2), and the amount of adsorbed metal (*Q*) with Equation (3) [24]:

(2) $$R=\frac{C_{0}-C}{C_{0}}\;\cdot 100,$$

(3) $$Q=\frac{\left(C_{0}-C\right)\cdot V}{W},$$

where *C_0_* is the initial concentration of the individual pollutant in wastewater after dyeing (mg/L); *C* is the concentration of pollutant after adsorption trial (mg/L); *Q* is the amount of adsorbed pollutant per weight of adsorbent (mg/g); *V* is the initial volume of wastewater (L); and *W* is the weight of adsorbent (g).

## 3. Results and Discussion

### 3.1. Colourimetric Evaluation of the Dyed Samples

**Figure 2:**
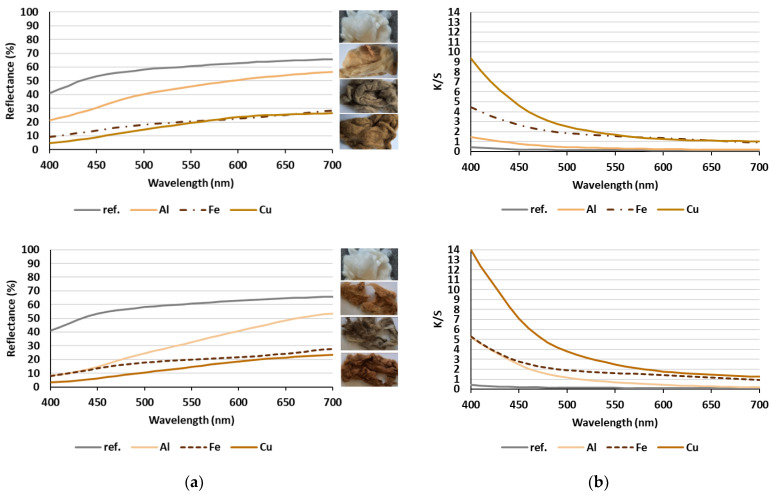
Colorimetric evaluation of un-dyed (ref.) and dyed wool fibers using different mordants: (**a**) Reflectance values and photographs of dyed samples; (**b**) K/S values; lower mordant concentration (**above**) and higher mordant concentration (**below**).

With the aim to study the coloration ability/color intensity of common walnut leaves’ extract for dyeing of wool fibers depending on different concentration and type of applied metallic salts (meta-mordanting), all samples were evaluated colorimetrically by measuring the reflectance in a visible spectrum (400–700 nm), from which the color strength (K/S values) were calculated according to Equation (1), as well as the color coordinates determined, i.e., lightness (L*), red/green axis (a*), yellow/blue axis (b*), chroma (C*) and hue (*h*), in all three directions of the CIE color space. The obtained results are gathered in Figure 2 and Table 1.

**Figure 3:**
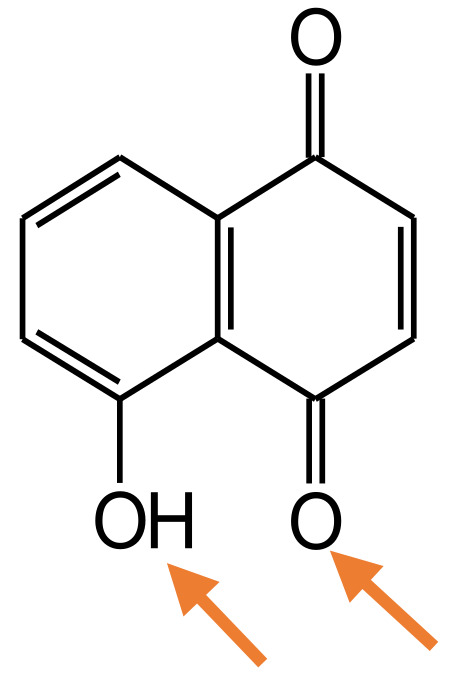
Chemical structure of 5-hydroxyl-1,4-naphthoquinone (juglone) with two active sites for metal-based complex formation.

Figure 2a shows the reflectance curves of the undyed (ref.) and dyed wool fibers without typical reflectance minimum as compared to the synthetic coloration. The application of walnut leaves’ extracts reduced the reflectance significantly and enhanced the K/S values over the entire visible spectrum (Figure 2b) as compared to the reference wool sample, regarding the concentration and the type of mordant used. The admixture of mordant in walnut leaves’ extract dyebath resulted in the formation of Al-, Fe- or Cu-complexes with 5-hydroxyl-1,4-naphthoquinone through the oxygen atom of the 1-hydroxy group and the quinone oxygen atom—active sites in Figure 3, and after that with free amino and carboxyl groups of wool fibers, leading to the fixation of natural dye as described in detail in [6,25].

Moreover, the presence of H^+^ ions due to the acidic dyebath causes the protonation of the amide linkage of wool and, thus, higher interactions with coloring compounds and enlarged color depth [13]. The *h*, C* and L* values are also affected significantly by the type of mordant, as can be perceived from Table 1. Generally, an FeSO_4_ mordant gives a bluer and less saturated color on a dyed sample, and CuSO_4_ mordant, the darkest shade in comparison with the other two mordants, regardless of the mordant concentration. Similar variations in color were reported by different authors [21,22,23,24,25,26]. By increasing the concentration of mordant, the color strength (K/S) increased and L* decreased, although these changes were not linear. The major difference in coloration was observed between samples dyed using an Al-based mordant.

### 3.2. Characterisation of the Adsorbents

**Figure 4:**
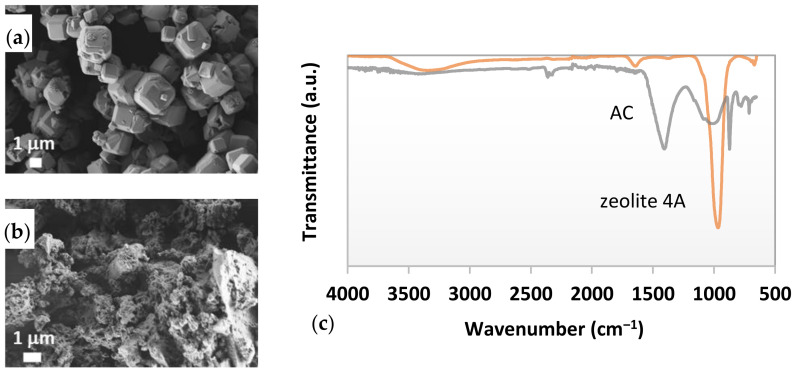
SEM images of: (**a**) 4A; (**b**) AC; and (**c**) FTIR spectra of the selected adsorbents.

Different analytical techniques were employed to provide some qualitative information about the structural and physical-chemical properties of the proposed adsorbents that could influence their treatment efficiency further. Those features are highly dependent on the type of feedstock for the preparation, as well as the methods and operational parameters during processing, i.e., hydrothermal crystallization (zeolite), carbonization/activation (activated carbon) [27] and further granulation [28]. With the aim of investigating the surface morphology of adsorbents, the SEM micrographs were taken and are presented in Figure 4a,b. In addition, the FTIR spectra were recorded from a wavenumber of 4000 cm^−1^ up to 650 cm^−1^ (Figure 4c) to examine the chemical composition on a molecular level that was essential for the physical or chemical interaction between adsorbents and pollutants in wastewater. Furthermore, the physical composition of adsorbents, i.e., specific surface area, micropore volume and pore size, was inspected using gas physisorption, since it is of great importance for their sorption capability [29].

The micrograph in Figure 4a shows unequivocally 4A crystallites embedded in both granulated forms (1–3 mm and 3–5 mm), with a well-defined three-dimensional cubic morphology and particles’ size ranging from 1 up to 3 μm. These results are consistent with the SEM of the synthesized zeolite 4A by Wang et al. [30]. Some spherical agglomerates of sodalite were also observed on the surface of 4A cubes, which usually occurred under hydrothermal synthesis as explained in [31]. Moreover, some impurities were perceived, probably on account of synthesis in an industrial manner. Additionally, Figure 4b reveals a heterogeneous, coarse-grained structure of AC with irregularly distributed pores of a high specific surface area, implying the good ability for adsorption of diverse pollutants.

From Figure 4c, typical peak positions for zeolites can be recognized on the FTIR spectra of the selected adsorbents, including the intensive transmittance peak at a low frequency area at a wavenumber of ca. 966 cm^−1^, due to the Si–O–Si and Al–OH stretching vibrations, a smaller peak at 681 cm^−1^ due to the Si-O-Al bending vibrations [24]. A small transmission band at ~1622 cm^−1^ is associated with the −OH hydrogen-bond bending vibration of zeolite 4A [30]. Additionally, the broad peak at 3650–3100 cm^−1^ is attributed to the stretching vibrations of the free hydroxyl groups [24]. The FTIR analysis of AC revealed two peaks at 1050 and 1136 cm^−1^, which were attributed to the vibration of C–O–C and C–O bonds, respectively [32]. The broad peak at 979 cm^−1^ was assigned to the aromatic ring vibrations, and at 1408 cm^−1^ to the aromatic C=O in carbonyl groups and C=C functional groups [27]. Mentioned bonds confirm the presence of carbonyl, aldehyde, lactone, and carboxyl groups onto the activated carbon surface as explained in [33].

The high BET specific surface area and total pore volume of AC, 840 m^2^/g and V_tot_ 0.575 cm^3^/g, respectively, revealed its highly porous structure and, therefore, high potential for adsorption of different compounds. On the other hand, the BET surface area of both zeolites 4A1 and 4A5 was rather low as compared to the AC, i.e., 80 and 105 m^2^/g, respectively. Rida et al. [29] reported similar results, concluding that low BET values indicated zeolites’ microporous structure with a relatively small internal surface.

### 3.3. Treatment of Wastewaters Utilising Adsorption

**Figure 5:**
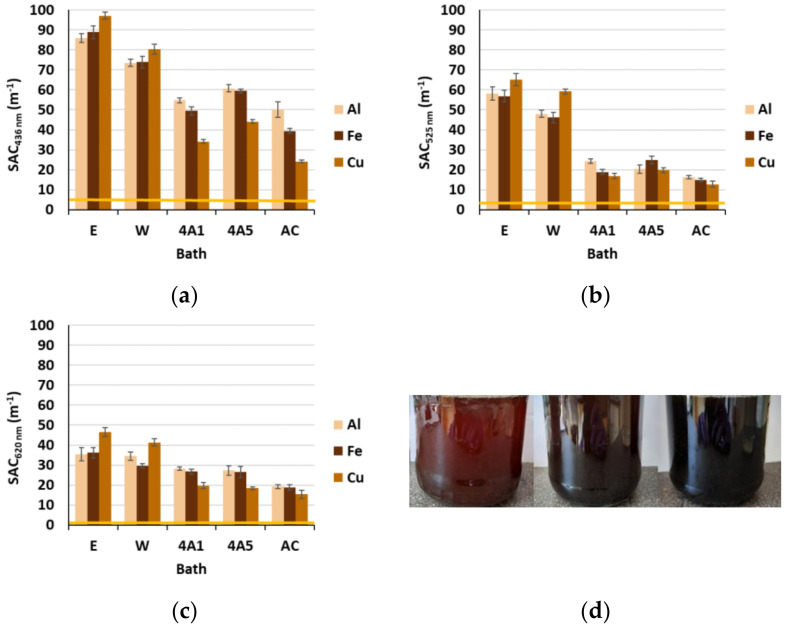
SAC values at: (**a**) 436 nm; (**b**) 525 nm; (**c**) 620 nm; and (**d**) corresponding photograph of effluents after dyeing. The yellow lines are the limit values for discharge of wastewater into watercourses from installations for the production, processing and treatment of textile fibers (UL RS 7/2007 and Council Directive 2006/11/ES).

**Figure 6:**
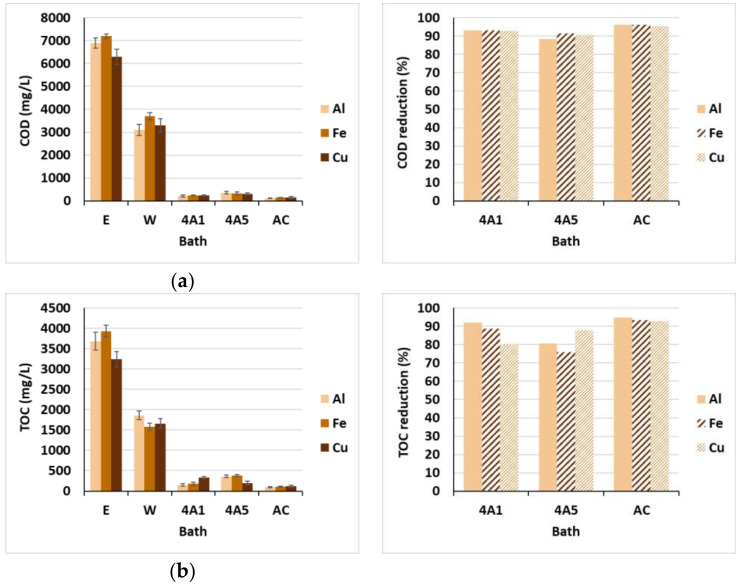
Pollution parameters in baths (**left**) and reduction in pollution (**right**): (**a**) COD; (**b**) TOC.

**Figure 7:**
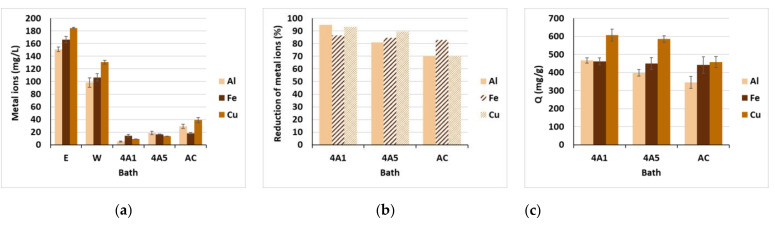
(**a**) Metal ions in baths in mg/L; (**b**) reduction in metallic ions in %; and (**c**) the amount of adsorbed metallic ions per weight of adsorbent (Q) in mg/g.

**Figure 8:**
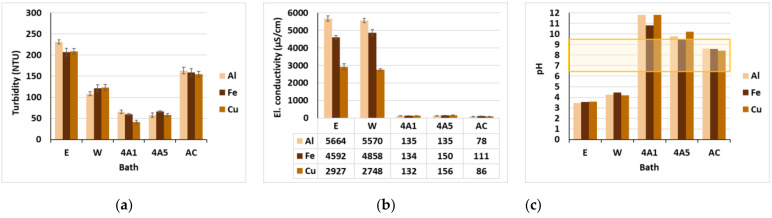
(**a**) Turbidity; (**b**) electrical conductivity; and (**c**) pH in baths.

A shake-flask adsorption trial was conducted employing commercially available adsorbents, i.e., synthetic zeolite 4A in two granulated forms (1–3 mm and 3–5 mm), and pelletized activated carbon for the treatment of wastewaters gained from the dyeing of wool with common walnut leaves’ extract, which were highly polluted with (colored) organic compounds and metallic salts, i.e., FeSO_4_, Al_2_(SO_4_)_3_ and CuSO_4_. Wastewaters with higher concentration (6 g/L) of individual salt were used for the adsorption trials. Zeolite was selected because of its specific porous structure composed of three-dimensional frameworks of SiO_4_ and AlO_4_ tetrahedra [34] and, thus, the ability to reduce diverse organic and inorganic ions, especially ions of heavy metals. Activated carbon was employed on account of its proven high adsorption capability to remove pollutants from various effluents. Adsorbents in the form of granulates or pellets can overcome some difficulties related to powdered form, e.g., losses of dust (enhanced turbidity), caking and lump formation, handling hazards, low surface-to-volume ratio and porosity, etc. [28]. The obtained results of the selected pollution parameters in Figure 5, Figure 6, Figure 7 and Figure 8 are marked by E-Al, E-Fe, E-Cu—exhausted baths included aluminum, ferrous or copper ions, and by W-Al, W-Fe, W-Cu—wastewaters after dyeing included aluminum, ferrous or copper ions. Abbreviations 4A1, 4A5 and AC mean treated wastewaters using granulated zeolite 4A in size 1–3 mm, granulated zeolite 4A in size 3–5 mm and pelletized activated carbon, respectively.

It can be noticed from Figure 5a–c that SAC values in wastewater after wool dyeing far exceeded the maximum values for discharge in watercourses as stated in UL RS 7/2007 and Council Directive 2006/11/ES at all three inspected wavelengths and, thus, need to be treated properly. The proposed adsorbents lowered wastewater coloration significantly, especially at a wavelength of 525 nm, although the SAC values remained highly above the tolerated values, as can also be perceived visually in Figure 5d, which could lead to low water transparency, affecting the photosynthesis of aquatic life. The best color reduction was achieved using AC (up to 78%), depending on the type of effluent/mordant and inspected wavelength. Such results were expected, since AC is a well-known and widely used adsorbent for discoloration of wastewaters including synthetic dyestuffs, due to its high surface area and high porosity [35,36]. A bit lower color reduction was achieved by employing 4A1 (up to 71%), followed by 4A5 (up to 66%). The adsorption of coloring compounds also depends highly on the pH of the effluent, since it causes a change in the surface charge of the adsorbent [24] as explained in detail below.

As can be seen from the left diagrams in Figure 6a,b, the initial COD and TOC values in dyeing baths were extremely high, due to the large amount of extracted (colored) organic compounds, significantly exceeding the maximum concentration for release in watercourses (UL RS 7/2007 and Council Directive 2006/11/ES), i.e., 200 mg/L (COD) and 60 mg/L (TOC). During dyeing, the organic compounds from the dyebath were exhausted to the fiber, as a result of a complex formation between the extract and mordant [3], which was reflected in the reduction in COD from 47.5% up to 55% and TOC from 48.8% up to 59.8%. In spite of the colorants’ good exhaustion, all three wastewaters (W) with different types of mordant were still highly polluted with organic matter, up to 3700 mg/L (COD) and up to 1860 mg/L (TOC), which are in accordance with the COD results determined by [20] for mahogany and myrobalan extracts, and by [21] for *C. urucurana* bark extract, and the TOC results obtained by [3] using henna extract. After the adsorption trials, organic pollution decreased drastically using all three adsorbents, irrespective of the type of metallic salt presented in the effluent. As can be seen from the right diagrams in Figure 6a,b, the COD reduction was up to 96% and TOC up to 95%, although the treated effluents still somewhat exceeded the limit values for discharge in watercourses. In order to lower the organic pollution, a higher amount of adsorbents should be added or some other parameters modified during adsorption, e.g., temperature, pH, addition of electrolyte, etc. Study of the adsorption kinetic and isotherm was not the aim of the presented research, but it will be examined closely in further work.

As can be seen from Figure 7a, the addition of mordants in walnut leaves’ extract influenced the extremely high values of Al^3+^, Fe^2+^ and Cu^2+^ ions in individual dyebaths. As already mentioned, metallic ions form a complex with juglon during wool dyeing, which enhances the exhaustion of the coloring compound, as well as enlarges its fixation strength and, consecutively, improves the color fastness properties of dyed fibers against light and washing. Nevertheless, a lot of unfixed metallic ions remained in the effluents after dyeing, i.e., 98.5 mg/L of Al^3+^, 106.6 mg/L of Fe^2+^ and 130.6 mg/L of Cu^2+^, which is in line with a study reported by [21]. The best reduction in metallic ions was achieved using the 4A1 zeolite (up to 94.7%) followed by 4A5 (89.6%), on account of their superior ion exchange and adsorption ability as compared to AC [34]. Furthermore, the initial wastewater’s pH has a significant role in the efficient adsorption of metallic ions, as explained by [24], since it determines the degree of the adsorbate ionization and the surface charge of the adsorbent. In our study, the pH of wastewaters was around pH 4.2 (Figure 8c), implying that metals exist in their cationic state and, thus, tend to be more soluble and mobile [22]. Moreover, in acidic pH, the zeolite surface is covered with Al–OH^2+^ and Si–OH^2+^ protons [37], with which the Al^3+^, Fe^2+^ and Cu^2+^ ions compete for the adsorption sites, increasing the zeolite adsorption capacity for the metals. At the same time, the positively charged zeolite surface became suitable for adsorption of the anionic colorants through electrostatic interaction [24], reducing the SAC values, as already mentioned (Figure 5). In a similar situation, wastewater pH/metallic ion adsorption dependency can be observed for AC, in which the surface became protonated under acidic conditions. Kolodynska et al. [27] reported the most efficient removal of metal ions at a pH above 5.

The turbidity of common walnut leaves’ extract in dye bath I was from 207 up to 231 NTU (Nephelometric Turbidity Unit) and in wastewater after dyeing (W) from 108 up to 123 NTU, as can be seen from Figure 8a. Turbidity is a criterion for light transmission through the water, and depends on the presence of colloidal particles and fine suspended solids [21]. In our study, it was caused mainly by organic compounds extracted from common walnut leaves. Turbidity in effluents was reduced from 40% up to 66% after treatment utilizing zeolites, regardless of the granulates size, due to the adsorption of the coloring compounds. On the other hand, treatment with AC enlarged turbidity, probably on account of the small, fragmented carbon particles which originated during shaking, resulting in a darker coloration of the wastewaters. Figure 8b shows high electrical conductivity in the dyebaths and effluents, which is attributed to the presence of metallic salts and other extracted, electrically charged compounds. It was significantly reduced after adsorption on all three adsorbents, as expected, since both 4A, as well as AC, have a high surface area and porous structure, and, thus, are well-known for their high ion exchange and adsorption capacity [34,38]. The extract of common walnut leaves had a pH near 5, but after the addition of mordants, the pH of the dyebaths was lowered to ~pH 3.6, as can be observed in Figure 8c. pH levels were increased slightly in all effluents up to ~pH 4.2 as compared to the dyeing baths; therefore, they need to be neutralized to pH 6.5–9.5 before discharge into watercourses. On the other hand, acid pH of wastewaters is essential for the efficient adsorption of metal ions by zeolites and AC as discussed in detail above (Figure 7). The enlargement of wastewater’s pH during absorption trials to pH 11.3 (4A1), pH 9.8 (4A5) and pH 8.5 (AC) was caused by the intrinsic pH of the individual adsorbents, as explained in [22].

## 4. Conclusions

In the first part of this study, the natural coloring compounds were extracted from dried common walnut (*Juglans regia*) leaves and used further for exhaustion (meta-mordant) dyeing of wool fibers together with three different metallic salts (Al_2_(SO_4_)_3_, FeSO_4_ and CuSO_4_) in two concentrations (3 and 6 g/L). The dyed samples were evaluated colorimetrically (reflectance, K/S and CIE values) in order to evaluate the coloration ability/color intensity of the extract, depending on the concentration and type of applied metallic salts. The obtained results proved that a higher concentration of mordant increased K/S and decreased lightness, although these changes were not linear. Generally, an FeSO_4_ mordant gives a bluer and less saturated color on a dyed sample, and CuSO_4_ the darkest shade in comparison with the other two mordants, regardless of the mordant concentration.

In the second part of the study, the colored effluents with 6 g/L of individual metallic salt were treated by utilizing a shake-flask adsorption approach. For this purpose, different adsorbents were selected and characterized qualitatively, i.e., granulated zeolite 4A in two particle sizes and pelletized AC, and, after that, employed in an adsorption trial for the potential reduction in color, COD, TOC, metals’ pH, turbidity and electrical conductivity. Some conventional terms, such as temperature, pH, time, addition of electrolyte, etc., were not changed during the trials since the main purpose of this research was the treatment of effluents as originated after natural wool dyeing. The gained results proved sufficient removal of exceeded metallic ions using 4A1 (up to 94.7%), followed by 4A5 (89.6%), as well as a reduction in turbidity and electrical conductivity, on account of the zeolite’s superior ion exchange capability as compared to AC. On the other hand, AC was more efficient for the reduction in organic pollution, COD up to 96% and TOC up to 95%, on account of its higher specific surface area and total pore volume and, therefore, showed higher potential for adsorption of different compounds in comparison to 4A. All three proposed adsorbents lowered wastewaters’ coloration remarkably, depending on the type of effluent/mordant and inspected wavelength, although the spectral absorbance coefficient (SAC) values remained high above the limit values for discharge of wastewaters into watercourses. Generally, wastewaters from natural dyeing of wool fibers with common walnut leaves’ extract were highly polluted with colored organic compounds, i.e., COD was up to 7200 mg/L and TOC up to 3930 mg/L, which could lead to low water transparency and high organic pollution, affecting the aquatic life negatively. Moreover, the considerable amounts of metallic salts are usually needed for sufficient dye fixation in the process, leading to the accumulation of disposed unfixed toxic metallic ions in the environment after dyeing. In the presented study, 98.5 mg/L of Al^3+^, 106.6 mg/L of Fe^2+^ and 130.6 mg/L of Cu^2+^ remained in wastewaters, which significantly exceeded the maximum concentrations permissible in a discharge as stated in EU Directive (Council Directive 2006/11/ES). The treatment approach utilizing zeolites as adsorbents offers an efficient, simple and low-cost solution to reduce the pollution of waters caused by natural dyeing, although it should be properly planned. Therefore, further work is needed, optimizing the amount of adsorbent, temperature, pH, time, electrolyte addition, etc., to enhance the wastewater’s treatment performance. Another aspect worth considering is the reuse of wastewaters after natural dyeing with a detailed characterization of colored materials, as well as wastewater analysis after each dyeing cycle in combination with wastewater treatment.

## Figures and Tables

**Table 1:** CIE color values of reference (ref.) and dyed samples using different concentrations (Conc.) and types of mordant.

Sample	Conc. (g/L)	L*	a*	b*	C*	*h*	Conc. (g/L)	L*	a*	b*	C*	*h*
Ref.		82.10	−0.11	7.46	7.46	90.84		82.10	−0.11	7.46	7.46	90.84
Al_2_(SO_4_)_3_	3	73.21	0.96	18.31	18.33	87.01	6	63.95	6.11	30.39	31.00	78.64
FeSO_4_	3	52.24	1.08	13.65	13.70	85.47	6	51.51	0.47	13.66	13.66	88.04
CuSO_4_	3	50.83	3.64	24.30	24.57	81.49	6	45.10	5.21	24.80	25.35	78.15

## Data Availability

Not applicable.

## References

[1] Vankar P.S., Shukla D. (2019). New Trends in Natural Dyes for Textiles.

[2] Pavithra K.G., Senthil Kumar P., Jaikumar V., Sundar Rajan P. (2019). Removal of colorants from wastewater: A review on sources and treatment strategies. J. Ind. Eng. Chem..

[3] Sharma A., Kadam S., Mathur P., Shahid-ul I., Sheikh J. (2019). Re-using henna natural dyeing wastewater for coloration and multifunctional finishing of linen fabric. Sustain. Chem. Phar..

[4] Chao Y.-C., Ho T.-H., Cheng Z.-J., Kao L.-H., Tsai P.-S. (2017). A study on combining natural dyes and environmentally-friendly mordant to improve color strength and ultraviolet protection of textiles. Fibers Polym..

[5] Haji A., Naebe M. (2020). Cleaner dyeing of textiles using plasma treatment and natural dyes: A review. J. Clean. Prod..

[6] Ojstršek A., Fakin D. (2019). Natural Dyeing of Wool Using Junglans regia (Common Walnut) Leaf Extract. Tekstilec.

[7] Amutha K., Grace Annapoorani S., Sudhapriya N. (2020). Dyeing of textiles with natural dyes extracted from Terminalia arjuna and Thespesia populnea fruits. Ind. Crops Prod..

[8] Varjani S., Rakholiya P., Ng H.Y., You S., Teixeira J.A. (2020). Microbial degradation of dyes: An overview. Bioresour. Technol..

[9] Arora J., Agarwal P., Gupta G. (2017). Rainbow of Natural Dyes on Textiles Using Plants Extracts: Sustainable and Eco-Friendly Processes. Green Sustain. Chem..

[10] Dias M., Valerio A., de Oliveira D., Ulson de Souza A.A., de Souza S. (2020). Adsorption of natural annatto dye by kaolin: Kinetic and equilibrium. Environ. Technol..

[11] Shams-Nateri A. (2011). Reusing wastewater of madder natural dye for wool dyeing. J. Clean. Prod..

[12] Haji A. (2019). Application of D-optimal design in the analysis and modelling of dyeing of plasma-treated wool with three natural dyes. Colorat. Technol..

[13] Arifeen W.U., Rehman F.U., Adeel S., Zuber M., Ahmad M.N., Ahmad T. (2021). Environmental friendly extraction of walnut bark-based juglone natural colorant for dyeing studies of wool fabric. Environ. Sci. Pollut. Res. Int..

[14] Tkaczyk A., Mitrowska K., Posyniak A. (2020). Synthetic organic dyes as contaminants of the aquatic environment and their implications for ecosystems: A review. Sci. Total Environ..

[15] Donkadokula N.Y., Kola A.K., Naz I., Saroj D. (2020). A review on advanced physico-chemical and biological textile dye wastewater treatment techniques. Rev. Environ. Sci. Bio/Technol..

[16] Wang Y., Wang H., Wang X., Xiao Y., Zhou Y., Su X., Cai J., Sun F. (2020). Resuscitation, isolation and immobilization of bacterial species for efficient textile wastewater treatment: A critical review and update. Sci. Total Environ..

[17] Deng D., Lamssali M., Aryal N., Ofori-Boadu A., Jha M.K., Samuel R.E. (2020). Textiles wastewater treatment technology: A review. Water Environ. Res..

[18] Samsami S., Mohamadizaniani M., Sarrafzadeh M.-H., Rene E.R., Firoozbahr M. (2020). Recent advances in the treatment of dye-containing wastewater from textile industries: Overview and perspectives. Proc. Saf. Environ. Prot..

[19] Somma S., Reverchon E., Baldino L. (2021). Water Purification of Classical and Emerging Organic Pollutants: An Extensive Review. ChemEngineering.

[20] Handayani W., Kristijanto A.I., Hunga A.I.R. (2018). Are natural dyes eco-friendly? A case study on water usage and wastewater characteristics of batik production by natural dyes application. Sustain. Water Resour. Manag..

[21] Dos Santos Silva P.M., Fiaschitello T.R., de Queiroz R.S., Freeman H.S., da Costa S.A., Leo P., Montemor A.F., da Costa S.M. (2020). Natural dye from Croton urucurana Baill. bark: Extraction, physicochemical characterization, textile dyeing and color fastness properties. Dyes Pigm..

[22] Ojstršek A., Gorjanc N., Fakin D. (2021). Reduction of Lead and Antimony Ions from the Crystal Glass Wastewaters Utilising Adsorption. Sustainability.

[23] Senthil Kumar P., Joshiba G.J., Femina C.C., Varshini P., Priyadharshini S., Arun Karthick M.S., Jothirani R. (2019). A critical review on recent developments in the low-cost adsorption of dyes from wastewater. Desal. Water Treat..

[24] Belachew N., Hinsene H. (2021). Preparation of Zeolite 4A for Adsorptive Removal of Methylene Blue: Optimization, Kinetics, Isotherm, and Mechanism Study. Silicon.

[25] Adem Ö., Özbek O., Vanglioglu F., Teker A.T., Boyraz D. (2021). Investigation of the dyeing properties of the colorant extracted from *Juglans regia L.* leaves on cellulosic and protein fabrics. J. Turk. Chem. Soc. A Chem..

[26] Choudhury A.K.R. (2018). Eco-friendly dyes and dyeing. Adv. Mat. Technol. Environ..

[27] Kołodyńska D., Krukowska J., Thomas P. (2017). Comparison of sorption and desorption studies of heavy metal ions from biochar and commercial active carbon. Chem. Eng. J..

[28] Charkhi A., Kazemeini M., Ahmadi S.J., Kazemian H. (2012). Fabrication of granulated NaY zeolite nanoparticles using a new method and study the adsorption properties. Powder Techn..

[29] Rida K., Bouraoui S., Hadnine S. (2013). Adsorption of methylene blue from aqueous solution by kaolin and zeolite. Appl. Clay Sci..

[30] Wang P., Sun Q., Zhang Y., Cao J. (2020). Effective removal of methane using nano-sized zeolite 4A synthesized from kaolin. Inor. Chem. Comm..

[31] Rios C.A., Williams C.D., Fullen M.A. (2009). Nucleation and growth history of zeolite LTA synthesized from kaolinite by two different methods. Appl. Clay Sci..

[32] Li H., Zheng F., Wang J., Zhou J., Huang X., Chen L., Hu P., Gao J.-M., Zhen Q., Bashir S. (2020). Facile preparation of zeolite-activated carbon composite from coal gangue with enhanced adsorption performance. Chem. Eng. J..

[33] Niazi L., Lashanizadegan A., Sharififard H. (2018). Chestnut oak shells activated carbon: Preparation, characterization and application for Cr (VI) removal from dilute aqueous solutions. J. Clean. Prod..

[34] Farghali M.A., Abo-Aly M.M., Salaheldin T.A. (2021). Modified mesoporous zeolite-A/reduced graphene oxide nanocomposite for dual removal of methylene blue and Pb^2+^ ions from wastewater. Inorg. Chem. Commun..

[35] Ho S. (2020). Removal of Dyes from Wastewater by Adsorption onto Activated Carbon: Mini Review. J. Geosci. Environ. Prot..

[36] Azari A., Nabizadeh R., Nasseri S., Mahvi A.H., Mesdaghinia A.R. (2020). Comprehensive systematic review and meta-analysis of dyes adsorption by carbon-based adsorbent materials: Classification and analysis of last decade studies. Chemosphere.

[37] Kobayashi Y., Ogata F., Saenjum C., Nakamura T., Kawasaki N. (2020). Removal of Pb^2+^ from aqueous solutions using K-type zeolite synthesized from coal fly ash. Water.

[38] Xie Z., Cheng J., Yan J., Cai W., Nie P., Chan H.T.H., Liu J. (2017). Polydopamine Modified Activated Carbon for Capacitive Desalination. J. Electrochem. Soc..

